# Cryptococcal Pleuritis in an Immunocompetent Patient: A Case Report and Literature Review

**DOI:** 10.7759/cureus.60260

**Published:** 2024-05-14

**Authors:** Ryuta Yamamoto, Kazunori Tobino, Shouta Sogabe, Yukari Saitou, Yumi Obata

**Affiliations:** 1 Respiratory Medicine, Iizuka Hospital, Fukuoka, JPN

**Keywords:** cryptococcus neoformans, immunocompetent, cryptococcal pleuritis, pleural effusion, cryptococcosis

## Abstract

Cryptococcosis, primarily an opportunistic infection, often occurs in immunocompromised patients but can also affect immunocompetent individuals. Cryptococcosis typically manifests in the lungs, but pleurisy is rare, particularly in immunocompetent patients. This report details a case of cryptococcal pleuritis in a 74-year-old immunocompetent male with a history of heart failure, presenting initially with pleural effusion. Diagnostic challenges arose due to the initial absence of intrapulmonary lesions. The diagnosis was eventually established through a surgical biopsy and tissue culture, revealing *Cryptococcus neoformans*. This case underscores the complexity of diagnosing cryptococcal infections, particularly in immunocompetent patients, and highlights the need for considering cryptococcosis in differential diagnoses of lymphocyte-predominant exudative pleural effusions.

## Introduction

Cryptococcosis is an opportunistic infection that occurs primarily in immunocompromised patients, but also in immunocompetent individuals [1]. Recent developments in immunosuppressive drugs and chemotherapy have increased the prevalence of opportunistic infections such as cryptococcosis, which is of high clinical significance, especially since cryptococcal infections in immunocompromised patients are associated with life-threatening diseases such as fungemia and meningitis. Cryptococcosis is caused by the encapsulated yeast-like fungus* Cryptococcus neoformans* or *Cryptococcus gattii*, which is widely distributed in the environment, especially in soil contaminated with bird droppings [2]. This fungus enters the body by inhaling airborne spores, making the lungs the most common site of cryptococcal infection [2]. In immunocompetent hosts, infection is usually limited to the lungs and may present with asymptomatic or mild respiratory symptoms. However, in immunocompromised individuals, especially those with cellular immunodeficiency, such as HIV/AIDS patients, organ transplant recipients, and patients on corticosteroid therapy, infection can disseminate from the lungs to other organs, especially the central nervous system, causing life-threatening meningitis [3]. The chest imaging findings of pulmonary cryptococcosis are relatively difficult to diagnose because they depend on the patient’s immune status. In immunocompromised patients, pulmonary cryptococcosis usually presents as solitary or multiple nodules and may mimic lung cancer or other granulomatous diseases such as tuberculosis or sarcoidosis [4]. In immunocompromised patients, imaging findings are more variable and may include diffuse stromal infiltrates, alveolar consolidation, and cavitary lesions [5]. Cryptococcal pleurisy is very rare, although multiple case reports exist, and its frequency in cryptococcosis as a whole is unknown. Cryptococcal pleurisy is more likely to occur in immunocompromised individuals, and the results of the first study clearly state that pleural lesions in cryptococcal infections are more commonly observed in immunocompromised hosts than in healthy individuals [6]. Herein, we report a case of cryptococcal pleurisy in an immunocompetent patient experienced at our hospital. This case highlights the importance of considering cryptococcosis as a potential cause of pleural effusion, even in immunocompetent individuals.

## Case presentation

A 74-year-old man with a history of hypertension, chronic atrial fibrillation, and chronic heart failure presented with a gradual onset of dyspnea on exertion over the past month. His symptoms had progressively worsened, significantly limiting his daily activities. He denied any associated chest pain, cough, fever, or weight loss. His primary care physician ordered a chest X-ray, which revealed a right-sided pleural effusion, leading to a referral for further evaluation at our hospital.

On initial examination, the patient appeared comfortable at rest. His vital signs included a blood pressure of 130/80 mmHg, a heart rate of 72 beats per minute, a respiratory rate of 18 breaths per minute, and an oxygen saturation of 96% on room air. A physical examination revealed decreased breath sounds over the right lower lung field without crackles or wheezes. No jugular venous distention or peripheral edema was observed.

**Figure 1:**
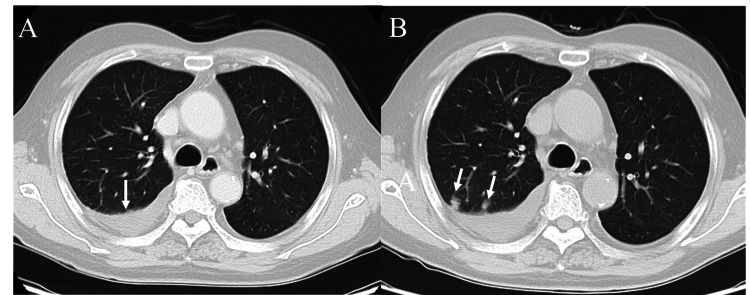
Chest CT scans before and after the development of pulmonary lesions (A) The initial chest CT scan demonstrates a right-sided pleural effusion (white arrow) without any evident lesions in the lung parenchyma. (B) A follow-up chest CT scan performed before the planned surgical pleural biopsy revealed the development of multiple new nodular opacities (white arrows) in the right upper lobe, suggesting an underlying infectious or malignant process.

A chest CT scan at the initial visit revealed a right pleural effusion without any pulmonary lesions (Figure 1A). Laboratory investigations showed a white blood cell count of 5,060/µL, total protein of 7.9 g/dL, lactate dehydrogenase (LDH) of 132 U/L, glucose of 135 mg/dL, and C-reactive protein of 0.04 mg/dL. Both blood T-SPOT.TB and serum mycobacterium avium complex antibody tests were negative.

Thoracentesis yielded a brownish, slightly turbid, lymphocyte-predominant (91%) pleural effusion. The pleural fluid analysis showed a pH of 7.53, adenosine deaminase (ADA) of 23 U/L, LDH of 66 U/L, protein of 3.7 g/dL, and glucose of 133 mg/dL. No microorganisms were found in the pleural fluid, and cytology did not reveal any malignant neoplasms or other distinctive findings.

A surgical right pleural biopsy was planned to achieve a definitive diagnosis. A follow-up preoperative chest CT scan disclosed multiple nodular shadows in the right upper lung lobe, indicative of a possible infectious or malignant process (Figure 1B). After the lung nodular shadows appeared, sputum culture was collected, but no significant bacteria were detected.

**Figure 2:**
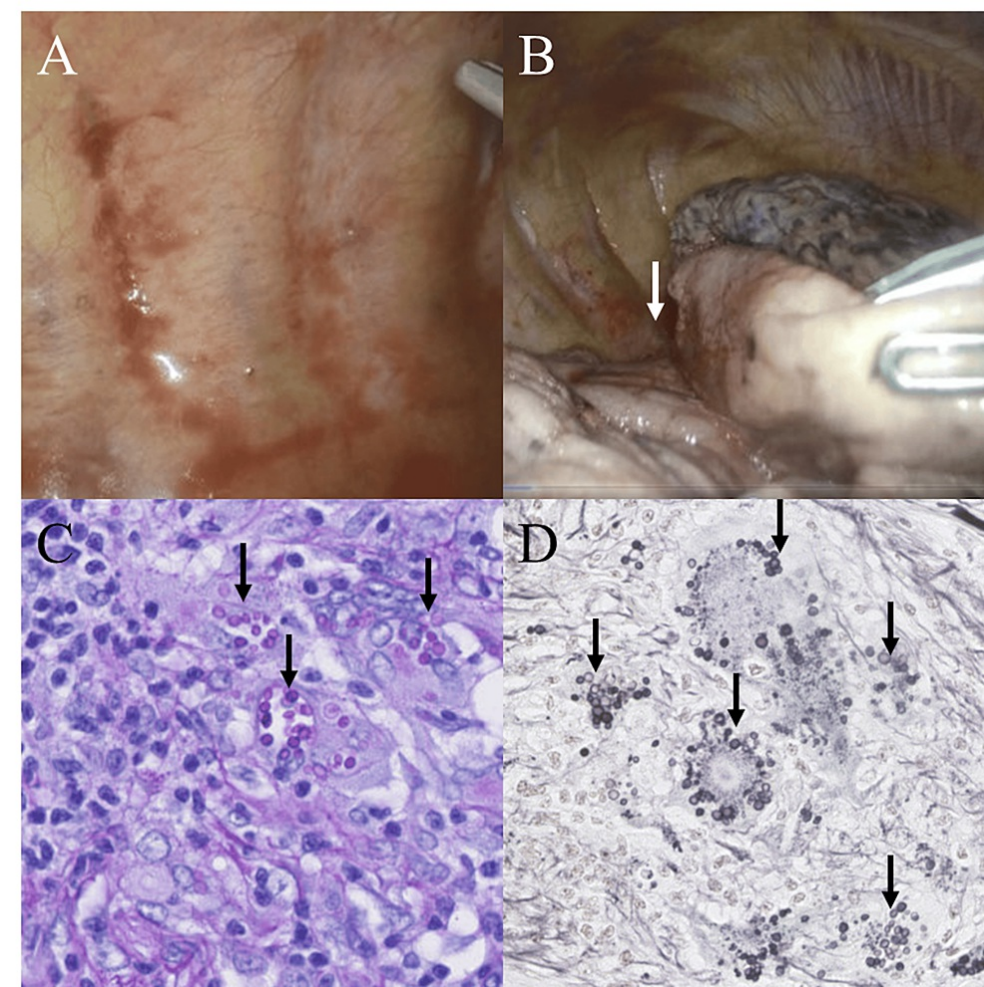
Thoracoscopic findings and histopathological examination of the resected lung nodule (A, B) Thoracoscopic examination of the right hemithorax reveals an absence of visible abnormalities on the parietal pleura. However, a small nodular lesion (white arrow) was observed in the interlobar region between the right upper lobe and middle lobe, corresponding to the radiographic findings. (C) Histopathological examination of the resected lung nodule with PAS staining demonstrates the presence of numerous yeast-like structures (black arrows), morphologically consistent with *Cryptococcus neoformans*. (D) Grocott’s methenamine silver staining further highlights the fungal elements (black arrows), confirming the diagnosis of pulmonary cryptococcosis. (Original magnification: C, 400x; D, 400x) PAS, periodic acid-Schiff

Consequently, the patient underwent a surgical right parietal pleural biopsy and a partial resection of the right upper lung lobe. Histological examination of the lung tissue, stained with periodic acid-Schiff and Grocott stain, confirmed yeast-like fungi consistent with Cryptococcus (Figure 2).

The tissue culture identified the fungus as *C. neoformans*. Histologic analysis of the parietal pleura showed only nonspecific inflammation, with no yeast-like fungi, and tissue culture did not grow any fungi. Serum cryptococcal antigen testing was negative, and screening tests for HIV and malignancy were also negative.

The patient was initiated on oral fluconazole (FLCZ) at a dose of 400 mg daily, which he tolerated well without adverse effects. Follow-up chest radiographs over the next six weeks showed a gradual resolution of the pleural effusion. The patient reported significant improvement in his dyspnea and was able to return to his normal daily activities. He completed a 24-week course of FLCZ and remained symptom-free at his six-month follow-up visit.

## Discussion

This is a rare case of cryptococcal pleurisy in an immunocompetent patient. Pleural effusion preceded the intrapulmonary lesions, and a thoracoscopic pleural and lung biopsy was required for a definitive diagnosis. In the absence of disseminated disease, monotherapy with FLCZ was effective.

Cryptococcus, characterized as a saprophytic, aerobic fungus with intracellular parasitic capabilities, has over 30 species, but only* C. neoformans* and *C. gattii *are recognized as pathogenic to humans [2]. Cryptococcosis predominantly occurs in immunocompromised patients, including individuals with malignancies, AIDS, transplant recipients, and those on immunosuppressive therapy. In East Asia, such as Japan and China, most cases of pulmonary cryptococcosis in immunocompromised patients were not AIDS related but associated with diabetes, collagen diseases, and cancer [7]. Cryptococcus is known to infect various organs, including the central nervous system, skin, blood, eyes, prostate, and bones. However, the lungs are the most common site of infection, as the infection is established by the inhalation of cryptococci [2]. The manifestation of pulmonary cryptococcosis varies widely, ranging from asymptomatic, isolated nodules to severe, life-threatening pneumonia, and is greatly influenced by the patient’s immune status. Radiographic findings in pulmonary cryptococcosis are diverse. In immunocompetent patients, CT often demonstrates well-defined nodules, which show F18-fluorodeoxyglucose uptake on positron emission tomography. These findings can be easily mistaken for lung cancer, leading to misdiagnosis in approximately 30% of such cases [7]. In contrast, chest imaging in immunocompromised patients frequently reveals infiltrative shadows, cavities, halo signs, and lymphadenopathy [8].

Cryptococcal pleurisy occurs mainly in immunocompromised patients. A review of 76 patients with pulmonary cryptococcosis revealed that five cases involved pleural effusions, with two of these patients being immunocompetent [9]. Our case is considered noteworthy due to cryptococcal pleuritis in an immunocompetent patient. Cases of cryptococcal pleuritis in immunocompetent individuals, where the clinical and laboratory details of the patients are described, are listed in Table 1.

**Table 1:** Case reports of cryptococcal pleuritis in immunocompetent patients 5FC, 5-fluorocytosine; ADA, adenosine deaminase; AMB, amphotericin B; FLCZ, fluconazole; ITCZ, itraconazole; L-AMB, liposomal amphotericin B; LDH, lactate dehydrogenase; ND, not described; VRCZ, voriconazole

Author	Age	Sex	Lesion side	Serum cryptococcal antigen	Comorbid lung lesions	Pleural effusion analysis							Diagnostic modality	Treatment
						pH	Protein (g/dL)	LDH (IU/L)	Differential cell counts	ADA (U/L)	Cryptococcal antigen	Culture		
Wu et al. [10]	29	Male	Left	Positive	Positive	ND	ND	ND	ND	ND	ND	ND	Transbronchial biopsy and percutaneous lung biopsy	FLCZ 400 mg/day, followed by VRCZ 400 mg/day
Izumikawa et al. [11]	24	Male	Right	Positive	Positive	ND	ND	444	Eosinophil: 13%	ND	Negative	Negative	Transbronchial biopsy and pleural biopsy	L-AMB→FLCZ→ITCZ→L-AMB→VRCZ with 5FC
Allena et al. [12]	50	Male	Left	ND	Positive	ND	ND	ND	Lymphocyte predominant	24.8	ND	ND	Transbronchial biopsy	FLCZ 400 mg/day for six months
Wong et al. [13]	30	Female	Bilateral	ND	Positive	ND	2.5	23	Polymorphonuclear cells: 91%	ND	Positive	Negative	Cryptococcal antigen test of the pleural fluid	AMB with 5FC therapy for 11 weeks, followed by oral FLCZ 400 mg/day
Rodríguez-Álvarez et al. [14]	78	Male	Right	Negative	ND	7.5	4.1	ND	Lymphocytes: 23%; polymorphonuclear cells: 39%	24.7	Not examined	Positive	Pleural fluid culture	VRCZ 400 mg/day
Chang et al. [15]	22	Male	Left	Positive	Negative	7	5.5	894	Lymphocytes: 37%; polymorphonuclear cells: 58%	ND	ND	Positive	Pleural fluid culture and a surgical lung biopsy	AMB 20 mg/day, followed by FLCZ 500 mg/day for 30 days
Our case	74	Male	Right	Negative	Positive	7.5	3.7	66	Lymphocytes: 91%	23.4	Not examined	Negative	Surgical lung and pleural biopsy	FLCZ 400 mg/day for six months

Pleural effusions in cryptococcosis are generally exudative, lymphocyte-predominant, and predominantly right sided. However, neutrophil-predominant, eosinophil-predominant, and transudative pleural effusions may also be seen [9]. Elevated ADA levels in pleural fluid have been reported, which may be diagnostic, but it is important to differentiate cryptococcal pleurisy from tuberculous pleurisy [16]. Detection of cryptococci in pleural fluid or pleural tissue is the gold standard for the diagnosis of cryptococcal pleurisy, but the sensitivity of pleural fluid culture is relatively low; Young et al. reported that pleural fluid culture was positive in 12 of 26 cases (46%) [17]. Because of the difficulty in diagnosing cryptococcal pleurisy by pleural fluid culture, measurement of cryptococcal antigen in pleural fluid, a less invasive method, should be considered as an option for the diagnosis of cryptococcal pleurisy. In a previous report on cryptococcal antigen testing in pleural fluid in cryptococcal pleurisy, 80% (8/10) of cases were positive, which was diagnostically useful even when pleural fluid cultures were negative [18]. Unfortunately, our patient was not tested for cryptococcal antigen in the pleural fluid. Antifungal treatment for cryptococcosis generally depends on the patient’s immunological status, underlying disease, and complications of meningoencephalitis and other disseminated lesions. Some cases of cryptococcal pleurisy in immunocompromised individuals have been treated with amphotericin as a disseminated disease, while others have been cured with FLCZ alone [12]. From the previously reported case reports, FLCZ monotherapy may be effective in cases of pleurisy alone, with no fungi present in the pleura or pleural effusion. Whether drainage should be performed depends on the individual case. In our patient, there were no lesions other than lung and visceral pleura, and the patient responded well to FLCZ monotherapy.

Cryptococcus is primarily a pulmonary pathogen, infecting the lungs first and then disseminating hematogenous material throughout the body. In our patient, the mechanism by which the pleural effusion preceded the intrapulmonary lesions is unknown, but it is possible that the microlesions in the lungs directly infiltrated the thoracic cavity. Also, since our patient was immunocompetent, it is possible that some local immune response abnormality allowed cryptococcal growth.

## Conclusions

This case highlights the importance of considering cryptococcosis as a potential cause of pleural effusion, even in immunocompetent individuals. A thoracoscopic biopsy may be useful for diagnosis in the presence of a preceding pleural effusion, and in the absence of disseminated disease, FLCZ monotherapy is the treatment of choice. Further case series are warranted to clarify the characteristics of cryptococcal pleurisy in immunocompetent patients and the treatment strategy.
